# Supplementary material for: Concentration-Dependent Pro- and Antitumor Activities of Quercetin in Human Melanoma Spheroids: Comparative Analysis of 2D and 3D Cell Culture Models
Source: Molecules. 2021 Jan 30;26(3):717. doi: 10.3390/molecules26030717 (PMC7866537; doi:10.3390/molecules26030717)
Supplement: Supplementary file 1 [file molecules-26-00717-s001.zip › molecules-1069028-supplementary-for proof/Supplementary_Figures_and_Videos/molecules-1069028-supplementary.docx]

**Supplementary Figure Descriptions**

**
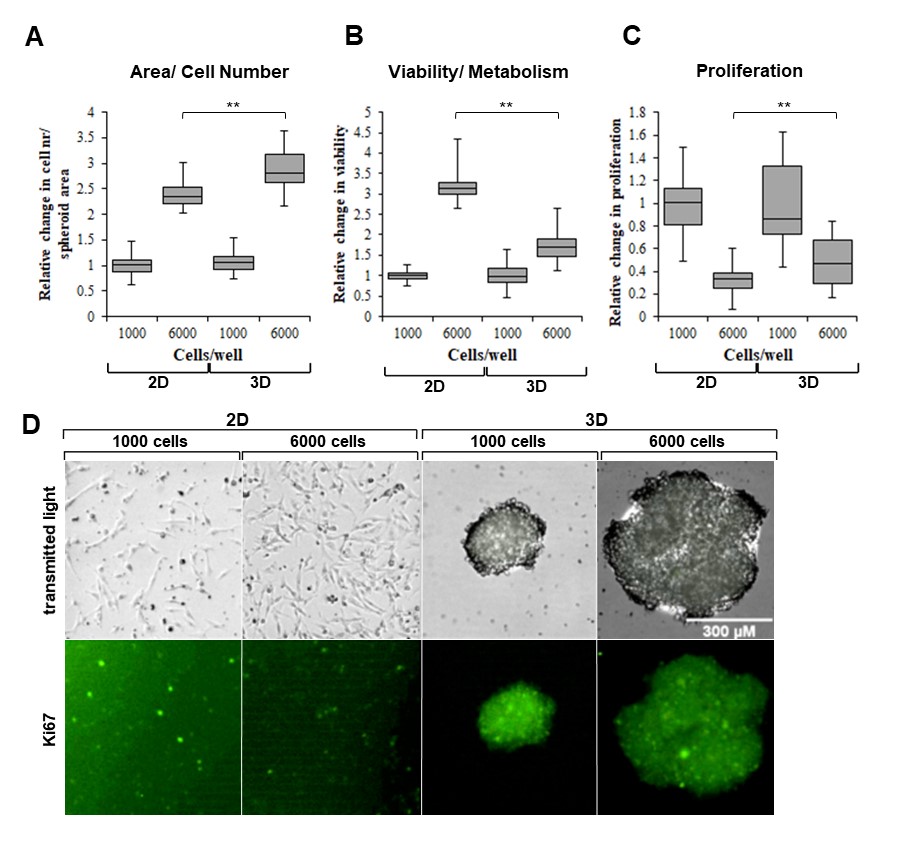
**

**Figure S1.** Strategy and validation of the high-content screening platform for evaluation of 2D and 3D cell cultures. 1000 or 6000 MCM DLN cells were cultured on flat plastic dishes (2D) or on non-adherent 96-well plates as 3D spheroids and different number of cells (2D), spheroid size, cell viability and proliferation compared, evaluated, and analysed 4 days later using the SpectraMax^®^ i3x Multi-Mode Microplate Reader with integrated Mini Max^TM^ 300 Imaging Cytometer (Molecular Devices, #5024062). (**A**) Cell count (object count feature) of single cells and area measurement of spheroids was performed. Bright field images (**D**) were segmented using the Fiji Macro INSIDIA and the area measured subsequently. (**B**) Metabolic viability was measured at Ex/Em 555/585 nm by using Presto blue assay according to the manufacturer’s instructions. (**C**) Proliferation was assessed on fixed and permeabilized cells stained with Ki-67 antibody (green) and cell tracker deep red (Ki-67-green/cell tracker). Fluorescence signals were graphically pictured, and its intensity was automatically computed. (**D**) Bright-field and fluorescence (Ki67) images from 2D and 3D cultures. Data were collected at least 3 times in octuplicates and normalized to the respective 1.000 cell control samples and expressed as box-whisker-plot with median (-), second and third quartile (25% of data greater or less than this value) and maximum and minimum whiskers with greatest or least value, excluding outliers.


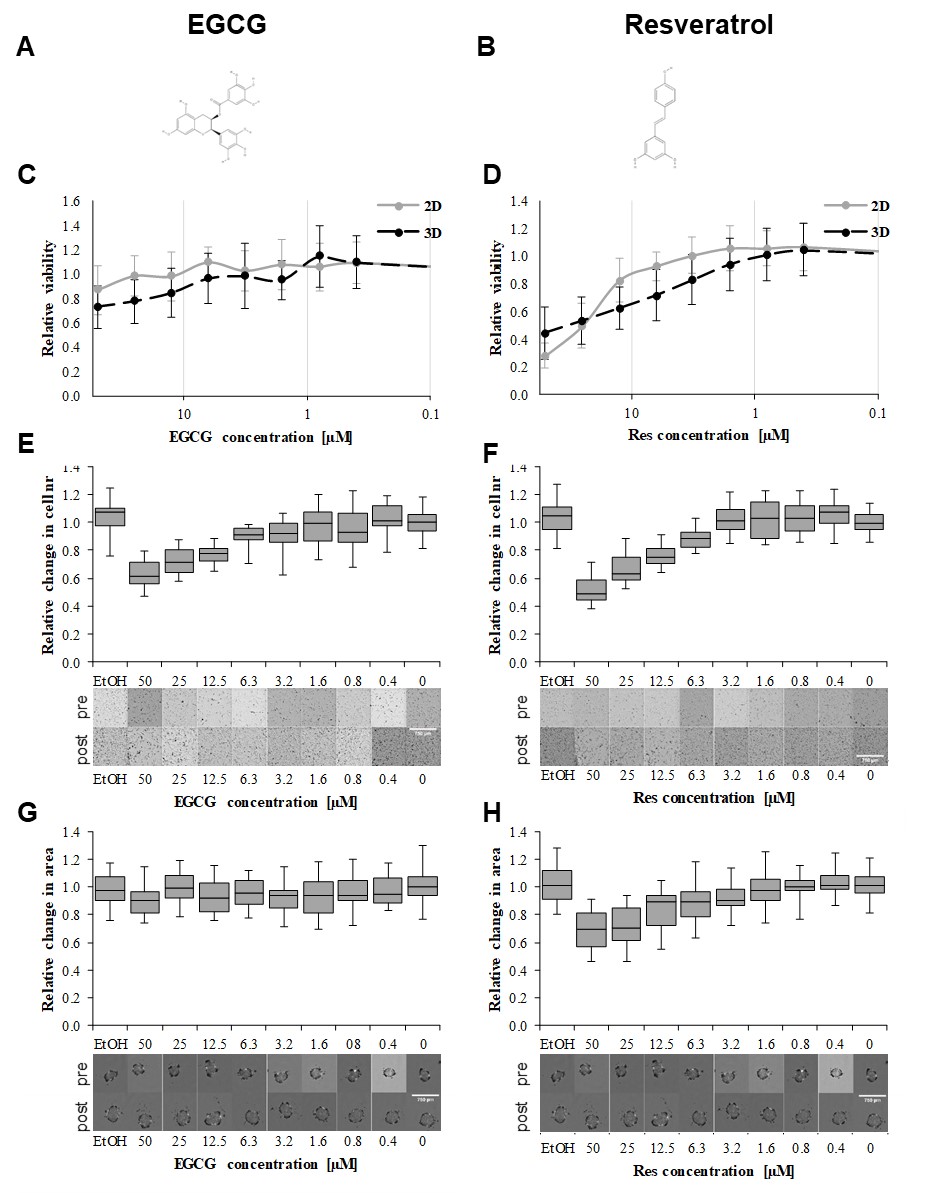


**Figure S2.** Effect of EGCG and resveratrol on cell viability and cell growth in 2D and 3D MCM DLN cells. 2D cell culture on plastic and melanoma spheroids on day 4 were treated with 50–0.4 µM of EGCG or resveratrol or left untreated and metabolic activity (viability), cell number (2D) and spheroid area evaluated 3 days later. Data are normalized to the equivalent solute concentration and to the non-treated cells. Chemical structures of (**A**) EGCG and (**B**) resveratrol. Timeline for 2D and 3D spheroid preparation, treatment and imaging. (**C** and **D**) Presto blue (cell viability) measurement in 2D and 3D human melanoma cell after treatment with (**C**) EGCG and (**D**) resveratrol. Data are expressed as mean ± S.D., performed at least 4 times in octuplicates. Evaluation of cell number (**E**, **F**) and spheroid area (**G**, **H**) of MCM DLN cells with EGCG (**E**, **G**) or resveratrol (**F**, **H**). Data are presented as box-whisker-plot (graphics) performed at least 4-times in octuplicates and corresponding images (below).


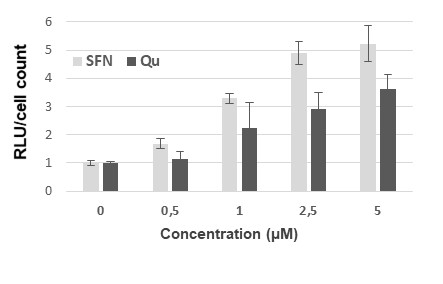


**Figure S3**. Evaluation of the Nrf2 luciferase reporter system. Nrf2 reporter were stable integrated into MCM DLN cells and reporter activity evaluated with the known Nrf2 activator sulforaphane (0 – 5 µM) and quercetin.

**Supplementary Video Descriptions**

**Video S1.** Phenotype of small spheroids. 1000 MCM DLN cells were cultured as three-dimensional (3D) cell aggregates using non-adherent plates. After 4 days, spheroids were fixed, permeabilized and stained with an antibody against the proliferation marker Ki-67 (green, top right), Hoechst (blue, top left) and phalloidin (red, bottom left). Images were analysed using a Leica TSC SP8 confocal laser scanning microscope. The bottom right shows all channels as a composite.

**Video S2.** Ki-67 distribution in small spheroids. The Z-stack of the 1.000 cell spheroid displayed in Video S1 is shown: Hoechst (blue, top left), Ki-67 (green, top right) (red, bottom left) and merged (bottom right). Ki-67 positive cells in spheroids fabricated with 1.000 cells are found evenly distributed.

**Video S3.** Phenotype of large spheroids. 6000 MCM DLN cells were cultured as three-dimensional (3D) cell aggregates using non-adhesion plates. After 4 days, spheroids were fixed, permeabilized and stained with an antibody against the proliferation marker Ki-67, Hoechst and phalloidin. Notably, Ki-67 positive cells are, in contrast to the smaller spheroids (Video S1), found only in the outer rim.

**Video S4.** Ki-67 distribution in large spheroids. The Z-tack of the 6000-cell spheroid displayed in Video S3 is shown: Hoechst (blue, top left), Ki-67 (green, top right) (red, bottom left) and merged (bottom right). Ki-67 positive cells in spheroids fabricated with 6000 cells are found only in the outer rim.
