# Supplementary figures and images for: Concentration-Dependent Pro- and Antitumor Activities of Quercetin in Human Melanoma Spheroids: Comparative Analysis of 2D and 3D Cell Culture Models
Source: Molecules. 2021 Jan 30;26(3):717. doi: 10.3390/molecules26030717 (PMC7866537; doi:10.3390/molecules26030717)

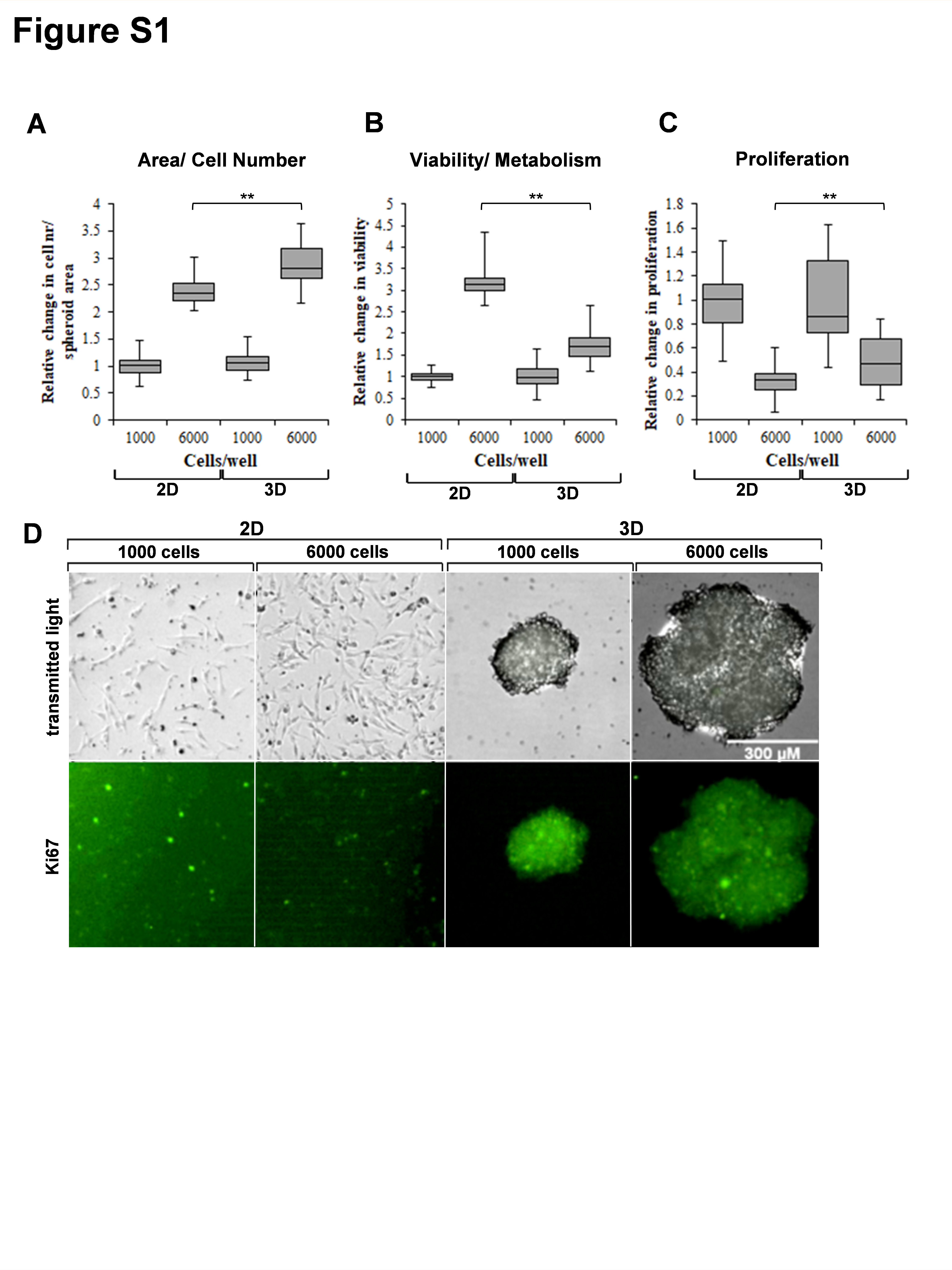

Supplement: Supplementary file 1 [file molecules-26-00717-s001.zip › molecules-1069028-supplementary-for proof/Supplementary_Figures_and_Videos/Fig.S1.tif]

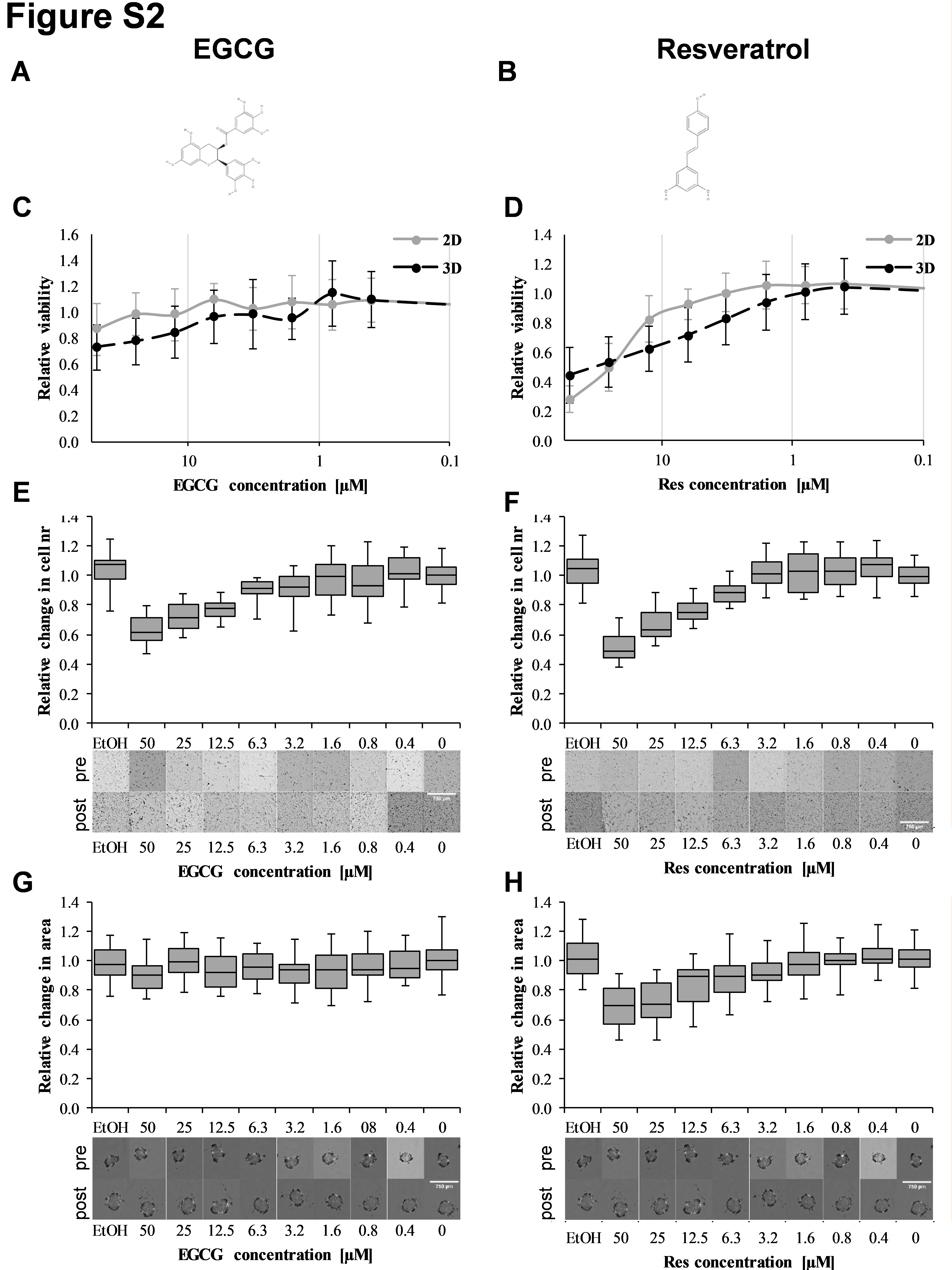

Supplement: Supplementary file 1 [file molecules-26-00717-s001.zip › molecules-1069028-supplementary-for proof/Supplementary_Figures_and_Videos/Fig.S2.tif]

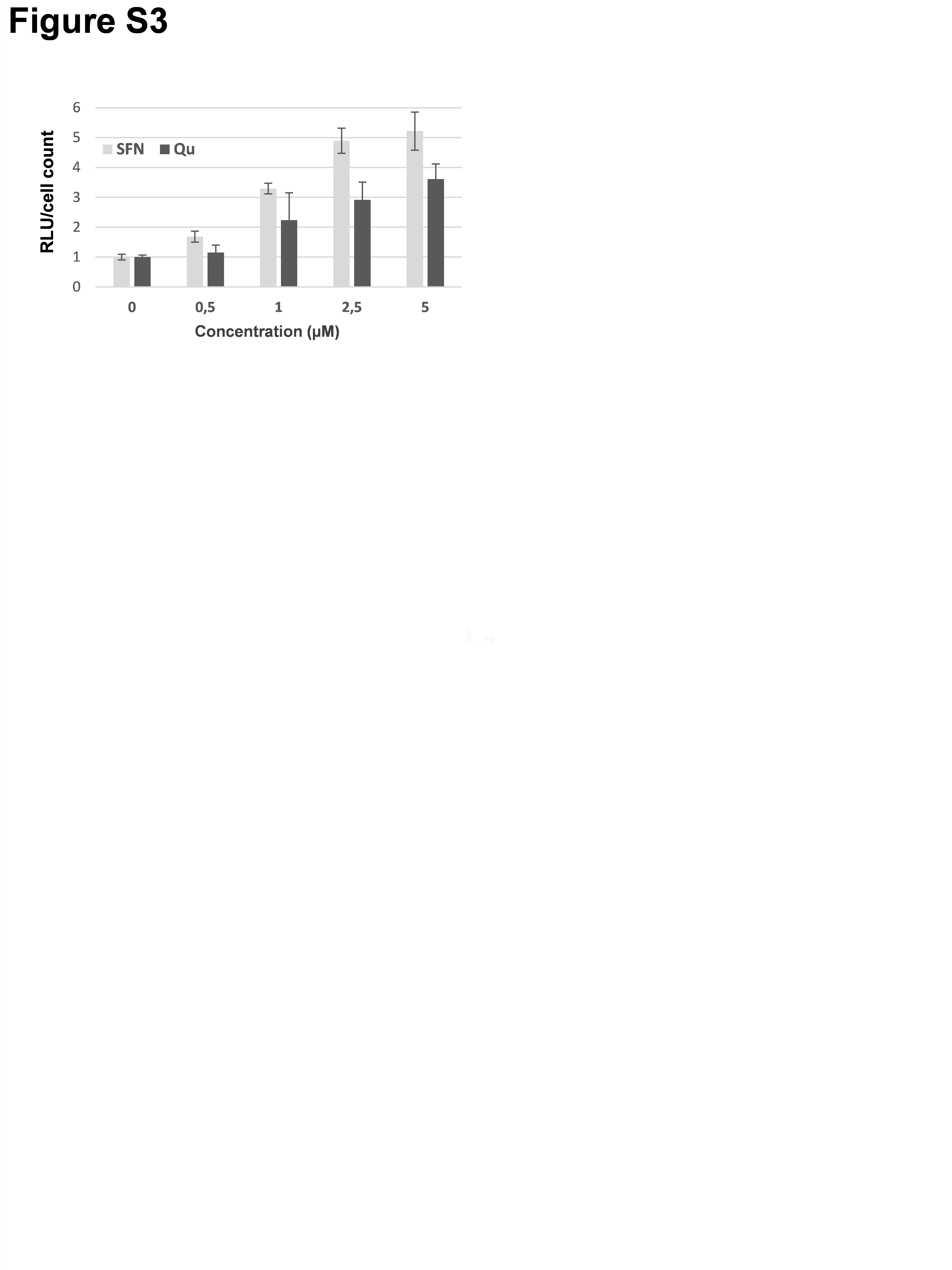

Supplement: Supplementary file 1 [file molecules-26-00717-s001.zip › molecules-1069028-supplementary-for proof/Supplementary_Figures_and_Videos/Fig.S3.tif]
